# Choline‐related‐inherited metabolic diseases—A mini review

**DOI:** 10.1002/jimd.12011

**Published:** 2019-01-25

**Authors:** Saskia B. Wortmann, Johannes A. Mayr

**Affiliations:** ^1^ University Childrens Hospital Paracelsus Medical University (PMU) Salzburg Salzburg Austria; ^2^ Institute of Human Genetics Technische Universität München Munich Germany; ^3^ Institute of Human Genetics, Helmholtz Zentrum München Munich Germany

**Keywords:** choline, epilepsy, fish odor disease, hereditary spastic paraparesis, uridine

## Abstract

In humans, the important water soluble, vitamin‐like nutrient choline, is taken up with the diet or recycled in the liver. Deficiencies of choline have only been reported in experimental situations or total parenteral nutrition. Currently, no recommended dietary allowances are published; only an adequate daily intake is defined. Choline is involved in three main physiological processes: structural integrity and lipid‐derived signaling for cell membranes, cholinergic neurotransmission, and methylation. Choline is gaining increasing public attention due to studies reporting a relation of low choline levels to subclinical organ dysfunction (nonalcoholic fatty liver or muscle damage), stunting, and neural tube defects. Furthermore, positive effects on memory and a lowering of cardiovascular risks and inflammatory markers have been proposed. On the other hand, dietary choline has been associated with increased atherosclerosis in mice. This mini review will provide a summary of the biochemical pathways, in which choline is involved and their respective inborn errors of metabolism (caused by mutations in *SLC5A7*, *CHAT*, *SLC44A1*, *CHKB*, *PCYT1A*, *CEPT1*, *CAD*; *DHODH*, *UMPS*, *FMO3*, *DMGDH*, and *GNMT*). The broad phenotypic spectrum ranging from malodor, intellectual disability, to epilepsy, anemia, or congenital myasthenic syndrome is presented, highlighting the central role of choline within human metabolism.

## 1 INTRODUCTION

The important water soluble, vitamin‐like nutrient choline (N,N,N‐trimethylethanolammonium), contains a quaternary ammonium group determining the cationic nature of the substance. It is the hydrophilic head group of the phospholipid lecithin (phosphatidylcholine [PC]). Humans take up choline with the diet (eg, beef liver, egg yolk, and cruciferous vegetables) or synthesize it in the liver and redistribute it from kidney, lung, and intestine.^1^ As de novo synthesis is possible, though complicated, it is not a vitamin in the strictest sense. Deficiencies of choline have not been reported in the general population, but have only been observed in experimental situations and in total parenteral nutrition.^2^, ^3^, ^4^, ^5^ Currently, no recommended dietary allowances are published by the European Food Safety Authority (EFSA), only an adequate daily intake is defined (eg, infants 180 mg/day and adults 400 mg/day) (EFSA Journal 2011;9[4]:2056 [23 pp.].). No cases of choline intoxication have been reported to date.

**Figure 1:**
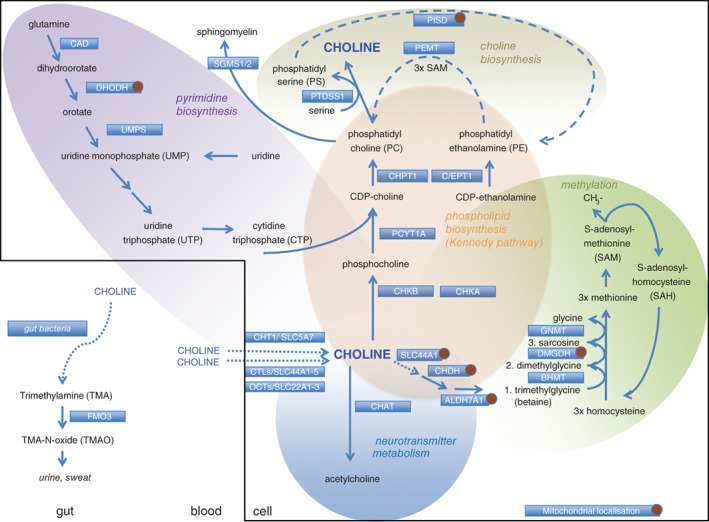
Choline‐related pathways. ALDH7A1, aldehyde dehydrogenase; BHMT, betaine‐homocysteine methyltransferase; CAD, carbamoyl phosphate synthetase/aspartate transcarbamoylase/dihydroorotase; CDP, cytidine diphosphate; C/EPT1, choline/ethanolamine phosphotransferase 1; CHAT, choline acetyltransferase; CHDH, choline dehydrogenase; CHKA, choline kinase alpha; CHKB, choline kinase beta; CHPT1, choline phosphotransferase 1; CHT1, high‐affinity choline transporter; CTL1, choline transporter‐like protein 1; DHODH, dihydroorotate dehydrogenase; DMGDH, dimethylglycine dehydrogenase; FMO3, flavin‐containing monooxygenases; GNMT, glycine N‐methyltransferase; OCTs, organic cation transporters; PCYT1A, choline‐phosphate cytidyltransferase A; PEMT, phosphatidylethanolamine N‐methyltransferase; PISD, phosphatidylserine decarboxylase; PTDSS1, phosphatidylserine synthase 1; SLC, solute carrier; UMPS, uridine monophosphate synthase

Choline is involved in three main physiological processes: structural integrity and lipid‐derived signaling for cell membranes, cholinergic neurotransmission, and methylation (as a major source for methyl groups via its metabolite trimethylglycine, Figure 1). Choline is recently gaining increasing public attention. This is based on observations that 77% of healthy men and 80% of postmenopausal women were shown to have low choline and subclinical organ dysfunction (eg, nonalcoholic fatty liver disease and muscle damage), which were resolved after 3 weeks of a choline rich diet.^6^ Furthermore, low choline has been related to neural tube defects in infants, even when the mother supplemented folate,^7^ and to stunting.^8^ Many other (small) studies speculate on further positive effects for some of the main adult health problems such as a positive effect on memory, a lowering effect on plasma homocysteine thereby lowering cardiovascular risks, lowering of inflammatory markers, decreased breast cancer risk, etc. (for overview: Reference 9). On the other hand, a choline‐ or carnitine‐rich diet resulted in increased formation of trimethylamine N‐oxide (TMAO) by gut bacteria in a mouse study and was associated with increased atherosclerosis.^10^

When considering the requirements for choline and methionine, one needs to take into account the close interrelationships with other methyl donors. Choline, methionine, and folate metabolism interact at the point that homocysteine is converted to methionine (Figure 1).

This mini review will provide a summary of the biochemical pathways in which choline is involved and their respective inborn errors of metabolism, highlighting the central role of choline in human metabolism without going into details for the disorder mentioned (Figure 1, Table 1). The methylation‐related disorders will only be mentioned with regards to the catabolism of choline, the classical methylation disorders will not be discussed.

## 2 UPTAKE OF CHOLINE INTO THE CELL AND FURTHER TRANSPORT INTO THE MITOCHONDRIA

Choline is taken up from the blood and transported via the plasma membrane by CHT1/SLC5A7, OCTs/SLC22A1‐3, and CTLs/SLC44A1‐5. SLC44A1, a member of the choline‐like transporter family, widely expressed in human tissues, is detected in both plasma and mitochondrial membranes and facilitates the choline uptake into the mitochondria, where the oxidation of choline to betaine takes place.^11^

## 3 CHOLINE AS PRECURSOR FOR DE NOVO NEUROTRANSMITTER BIOSYNTHESIS

Cholinergic neurons take up choline for acetylcholine synthesis by the high‐affinity choline transporter (CHT1, encoded by *SLC5A7*). Biallelic variants in *SLC5A7* underlie congenital myasthenic syndrome 20 (MIM #617143) characterized by muscular hypotonia and weakness, ptosis, poor sucking, and swallowing and prominent episodic apnea with a neonatal onset.^12^ Severity can vary, and acetylcholine esterase inhibitors show good results in some patients. Interestingly, heterozygous variants in the same gene lead to distal hereditary motor neuronopathy type VIIa (HMN7A, MIM#158580) with progressive distal muscle wasting and weakness affecting the upper and lower limbs from the second decade onwards. Additionally, the tenth cranial nerve is involved, which leads to vocal cord paresis.^13^ For both defects, no studies on treatment with choline have been published.

The biosynthesis of acetylcholine from choline is performed by choline acetyltransferase (CHAT, *CHAT*). Biallelic variants in this gene underlie congenital myasthenic syndrome 6 (MIM #254210).^14^ The clinical signs are comparable to congenital myasthenic syndrome 20, and most patients respond to treatment with acetylcholine esterase inhibitors.^15^

## 4 CHOLINE AS PRECURSOR FOR DE NOVO PHOSPHOLIPID BIOSYNTHESIS

Eugene Kennedy first identified the pathways predominant for de novo synthesis of PC (cytidine diphosphate [CDP]‐choline pathway) and phosphatidylethanolamine (CDP‐ethanolamine pathway) in mammals.^16^ Choline transporter‐like protein 1 (CTL1, *SLC44A1*) is believed to be the main choline transporter for the Kennedy pathway. SLC44A1 deficiency has been related to choline deficiency and membrane alterations in skin fibroblasts of a single patient with postural orthostatic tachycardia syndrome (no genetic data presented).^17^

Choline kinase alpha (CHKA, *CHKA*) and beta (CHKB, *CHKB*) phosphorylate choline. Deficiency of CHKB (autosomal recessive mutations in *CHKB*) was first described in a natural occurring mouse model showing a muscular dystrophy with a unique mitochondrial morphology in muscle fibres.^18^ Subsequently, the same phenotype was reported in humans.^19^ Single‐nucleotide polymorphisms in the CHKB locus have been associated with susceptibility to narcolepsy with cataplexy.^20^ No human phenotype has been associated to defective CHKA to date.

In the next step, phosphocholine is activated by the addition of cytidine triphosphate (CTP) and CDP is formed. This is the key rate‐limiting step performed by choline‐phosphate cytidyltransferase A (PCYT1A, *PCYT1A*), which is ubiquitously expressed, and by choline‐phosphate cytidyltransferase B (PCYTB) expressed in selected tissues. Biallelic variants in *PCYT1A* have been associated with cone‐rod dystrophy, either isolated or in combination with spondylometaphyseal dysplasia (MIM#123695).^21^, ^22^ Additionally, congenital lipodystrophy, fatty liver, severe insulin resistance, and diabetes were seen in two patients, providing evidence for an additional and essential role of PCYT1A‐generated PC in the normal function of white adipose tissue and insulin action.^23^ The final step from CDP choline to PC is performed by choline phosphotransferase 1 (*CHPT1*) or choline/ethanolamine phosphotransferase 1 (CEPT1, *CEPT1*). The latter has a dual specificity and also serves in the CDP‐ethanolamine biosynthesis, the second half of the Kennedy pathway. Recently, one pedigree with biallelic variants in *CEPT1* has been described with a complex neurological phenotype with hereditary spastic paraparesis, developmental delay, intellectual disability, dysarthria, retinal pigmentary abnormalities, and cone‐rod dystrophy.^24^

## 5 CHOLINE DE NOVO SYNTHESIS

Although complicated, choline can be synthesized de novo from PC as a secondary pathway. Phosphatidylserine synthase (PTDSS) 1 as well as 2 catalyzes the conversion of PC to Phosphatidyl serine (PS), thereby releasing choline. Additionally, 30% of choline in the liver is generated via phosphatidylethanolamine N‐methyltransferase. In mice, PTDSS1 and PTDSS2 can compensate for each other,^25^, ^26^ but simultaneous disruption of both genes is lethal, implying that PTDSS is absolutely required for viability.^25^ Dominant heterozygous mutations in *PTDSS1*, leading to a gain in enzyme function, have been associated to a syndrome of sclerosing bone dysplasia, intellectual disability and distinct craniofacial, dental, cutaneous (cutis laxa) and distal‐limb anomalies (Lenz‐Majewski syndrome [MIM #151050]).^27^

## 6 THE ROLE OF CHOLINE IN DE NOVO PYRIMIDINE METABOLISM

CTP is one of the end products of de novo pyrimidine synthesis and is a central component of the Kennedy pathway when converted to CDP and subsequently to CDP choline. Biallelic variants in the multienzyme complex CAD (carbamoyl phosphate synthetase 2, aspartate transcarbamylase, and dihydroorotase, *CAD*, MIM #616457) have recently been reported to lead to a progressive early infantile epileptic encephalopathy with dyserythropoietic anemia and tetraparesis. Anemia is a well‐known feature of another defect in the same pathway (uridine monophosphate synthase [UMPS] deficiency, *UMPS*, MIM #258900) and can be explained by the decreased red cell membrane stability due to lack of pyrimidines. One could speculate that the epileptic encephalopathy and other central nervous system findings are related to defective phospholipid biosynthesis.^28^ Importantly, uridine treatment leads to immediate cessation of seizures and regaining of lost skills. One could wonder if additional choline supplementation would also be beneficial for defects in de novo pyrimidine synthesis. Uridine supplementation was reported nonbeneficial in a dihydroorotate dehydrogenase (DHODH) defect (MIM #263750).^29^

## 7 THE ROLE OF CHOLINE AS METHYL DONOR

The oxidation of choline is irreversible, and formation of trimethylglycine (betaine) is performed in two steps by choline dehydrogenase (*CHDH*, no MIM entry) and aldehyde dehydrogenase (ALDH7A1, also known as antiquitin, *ALDH7A*). The latter is also an alpha‐aminoadipic semialdehyde dehydrogenase in the pipecolic acid pathway of lysine catabolism and leads to pyridoxine‐dependent epilepsy when deficient (MIM #266100).^30^ No deficiency of CHDH in humans has been reported to date. It is worth mentioning that two other disorders in the context of this article as the three enzymes—and their corresponding deficiencies—involved in the conversion of homocysteine to methionine, are not often mentioned in the context of methylation disorders. Betaine‐homocysteine methyltransferase (*BHMT*, MIM *602888) catalyzes the first step, here no corresponding human disorder is known. Dimethylglycine dehydrogenase (*DGMDH*) converts dimethylglycine into sarcosine. Deficiency has been reported in a single case: an adult with abnormal body odor resembling fish and elevated serum creatine kinase as well as muscle fatigability (MIM #605850).^31^, ^32^ While the malodor is seen in another defect related to choline (as detailed below), one cannot exclude that the other signs may be attributable to another disorder. How excessive dimethylglycine leads to fish odor is not described by the authors. Conversion of sarcosine to glycine is performed by glycine N‐methyltransferase (*GNMT*). Deficiency has been described in four patients (MIM #606664) with hepatomegaly and elevated transaminases and seems to be less severe than a knock out mouse model also showing hepatic glycogen storage, hypercholesterinemia, hypoglycemia, and low white blood cell count.^33^, ^34^, ^35^, ^36^

## 8 THE ROLE OF CHOLINE IN MALODOR

Gut microbiota specifically processes choline, PC, carnitine, and other dietary nutrients to produce trimethylamine (TMA). TMA is absorbed in the gut and converted in the liver to TMA N‐oxide (TMAO) by hepatic flavin‐containing monooxygenases (eg, FMO3). Autosomal recessive variants in *FMO3* (MIM #602079) were shown to underlie trimethylaminuria or fish odor syndrome.^37^ Affected individuals suffer from a strong, fishy body odor as the excess TMA is released in the person's sweat, urine, reproductive fluids, and breath. There are no other organic signs, but from a psychosocial perspective the condition can be devastating for affected individuals, attempted suicides have been reported.^38^, ^39^

## 9 CONCLUDING REMARKS

Choline is a central metabolite in human metabolism. Given its good bioavaibility after oral intake, more research is needed to identify the good effects on human health but also potential harmful side effects.

## AUTHOR CONTRIBUTIONS

Both authors jointly concepted and designed the study, analyzed and interpreted the data, drafted the article, and critically revised it for important intellectual content.
